# Supplementary material for: MV140 Mucosal Vaccine Induces Targeted Immune Response for Enhanced Clearance of Uropathogenic E. coli in Experimental Urinary Tract Infection
Source: Vaccines (Basel). 2024 May 14;12(5):535. doi: 10.3390/vaccines12050535 (PMC11126127; doi:10.3390/vaccines12050535)
Supplement: Supplementary file 1 [file vaccines-12-00535-s001.zip › vaccines-2971359-supplementary.pdf]

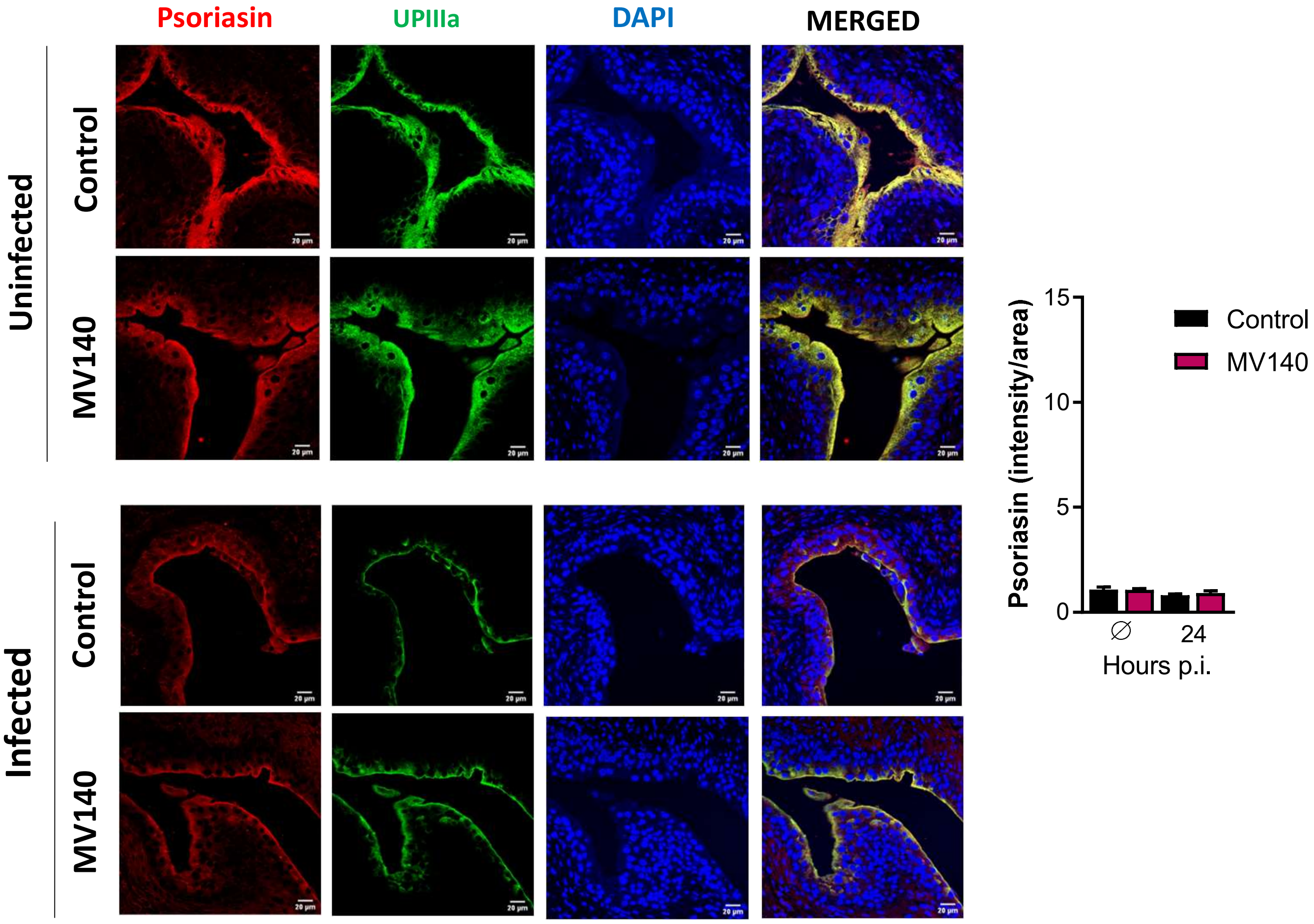

Supplementary Figure S1. Psoriasin protein expression in bladder urothelium at 24 hours post-infection, analyzed by immunofluorescence. Quantification of intensity per area from 3-5 random view fields is represented. Mean ± SEM of one (uninfected) or two (24 h p.i.) independent experiments is shown (n≥3). (A-C) Mice were immunized with control (vaccine excipients, black) or MV140 (magenta) and subsequently infected as stated in Figure 1A. Normal distribution was assessed using Shapiro-Wilk test. *P* values were calculated by unpaired Student's t-test comparing between treatment groups were found. Ø, uninfected; p.i., post-infection; UPIIIa, uroplakin IIIa.
